# Renal aging and its consequences: navigating the challenges of an aging population

**DOI:** 10.3389/fphar.2025.1615681

**Published:** 2025-07-24

**Authors:** Meiqi Zhang, Haifeng Ni, Yumeng Lin, Ke Wang, Tingke He, Lan Yuan, Zhongyu Han, Xiaohong Zuo

**Affiliations:** ^1^School of Medical and Life Sciences, Chengdu University of Traditional Chinese Medicine, Chengdu, China; ^2^Institute of Nephrology, Zhongda Hospital, Southeast University, Nanjing, China; ^3^Health Management Center, Nanjing Tongren Hospital, School of Medicine, Southeast University, Nanjing, China; ^4^ Deyang Hospital Affiliated Hospital of Chengdu University of Traditional Chinese Medicine, Deyang, China; ^5^College of Veterinary Medicine, Sichuan Agricultural University, Chengdu, China; ^6^Eye School of Chengdu University of Traditional Chinese Medicine, Chengdu, China; ^7^ Ineye Hospital of Chengdu University of Traditional Chinese Medicine, Chengdu, China; ^8^ Key Laboratory of Sichuan Province Ophthalmopathy Prevention and Cure and Visual Function Protection with Traditional Chinese Medicine Laboratory, Chengdu, China

**Keywords:** renal aging, AKI, CKD, DN, ESRD

## Abstract

With the aggravation of population aging, kidney aging and its impact on health have been widely concerned. Renal aging not only involves structural and functional changes but also is significantly linked to the occurrence and progression of some kidney diseases. Mechanisms of renal aging include oxidative stress, reduced Klotho levels, cellular senescence, and chronic inflammation. These changes lead to a sustained reduction in renal filtration, reabsorption, secretion, as well as endocrine function, which in turn affects overall health. Renal structural changes mainly include glomerulosclerosis, tubular degeneration and interstitial fibrosis. These structural changes are closely related to the decline of kidney function and may lead to the occurrence of chronic kidney disease (CKD). In addition, elderly individuals experience a higher rate of acute kidney injury (AKI) and face poorer prospects for recovery. The prevalence of age-related kidney disease, especially diabetic nephropathy (DN), increases with age. End-stage renal disease (ESRD) refers to the most advanced stage of CKD, in which the kidneys of patients show signs of premature aging compared with those of healthy people. Measures to prevent and delay renal aging in daily life, including healthy lifestyle, proper diet, and adequate exercise, are also discussed in this manuscript.

## 1 Introduction

Aging manifests in multiple systems throughout the entire body. Renal aging not only results in structural changes and functional decline of the kidney but is also linked to the onset and progression of some renal conditions. As age progresses, the kidney experiences structural transformations, such as glomerulosclerosis, tubular degeneration, interstitial fibrosis, and arteriosclerosis with narrowing of the blood vessels.

The aging process of kidneys is intimately linked to a heightened risk of developing kidney diseases. Elderly people have a greater vulnerability to acute kidney injury (AKI), and their recovery ability after AKI is poor due to the deterioration of renal function seen in aging. Renal aging is a significant contributor to the onset and progression of chronic kidney disease (CKD). Diabetes is a risk factor for CKD, and renal aging may aggravate the damage of diabetes to renal function. Renal aging increases the probability of CKD progressing to end-stage renal disease (ESRD). In addition, elderly patients face an increased likelihood of complications associated with renal transplantation.

In this manuscript, we delve into the processes underlying renal aging and detail the structural and functional alterations that accompany it. We also investigated the strong association of aging with a variety of renal diseases and related treatments.

## 2 Mechanisms of renal aging

**FIGURE 1:**
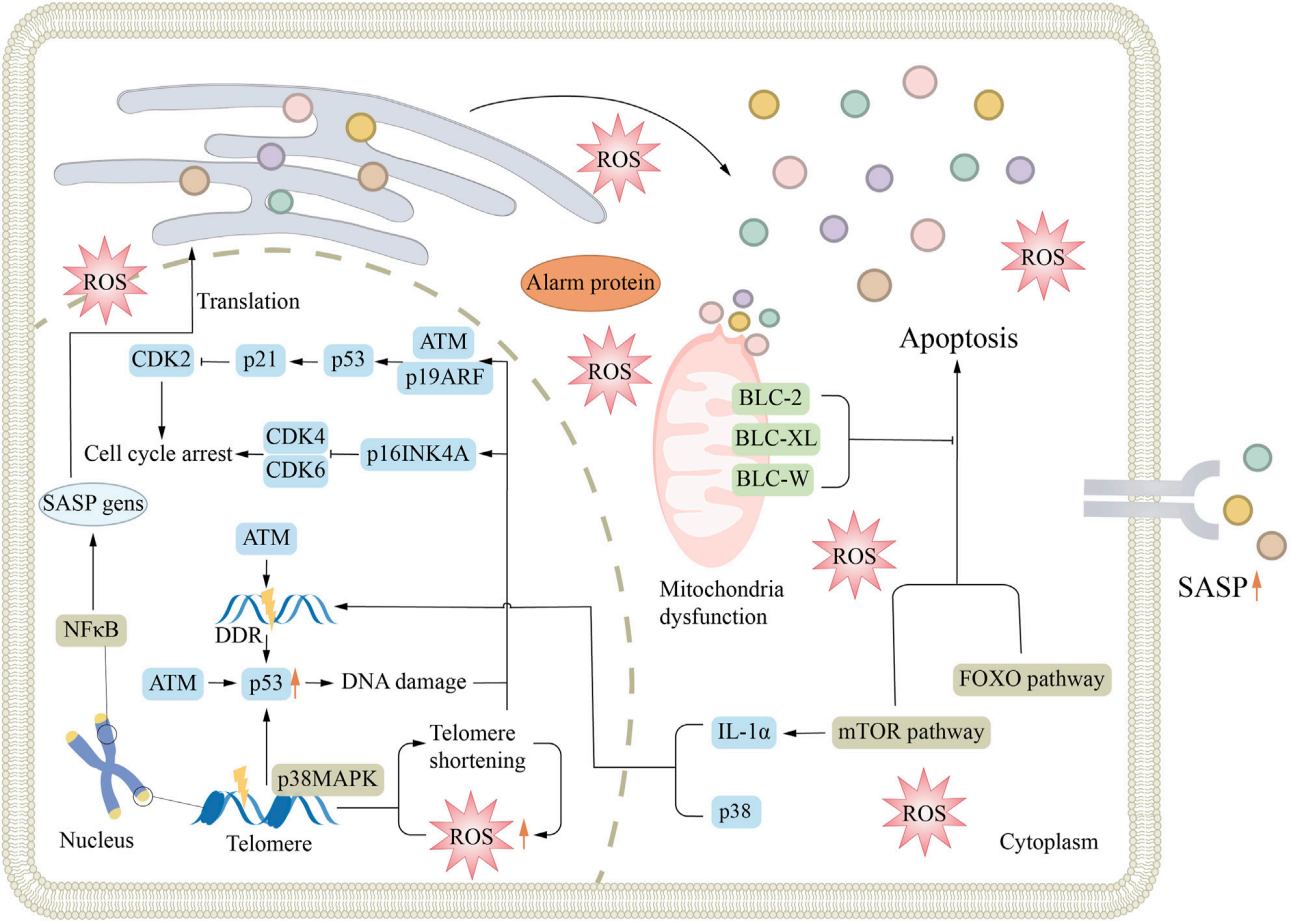
Mechanisms associated with renal aging. Aging is associated with many mechanisms, such as DDR, cellular senescence, oxidative stress, mitochondrial dysfunction, and signaling pathway activation, which all lead to cell division cycle arrest and promote the formation of senescence. ATM, ataxia telangiectasia mutation; DDR, DNA-damage response; ROS, reactive oxygen species; SASP, senescence-associated secretory phenotype.

The mechanisms of renal aging are intricate and mainly involve oxidative stress, decreased Klotho protein levels, cellular senescence, and chronic inflammation (Figure 1). Oxidative stress leads to the buildup of reactive oxygen species (ROS) in cells, resulting in damage of cell structure and function. The decrease of Klotho protein affects the repair function of the kidney. Cellular senescence reduces the regenerative capacity of renal tissue. Chronic inflammation further degrades renal function by continuously damaging cells and changing tissue structure. These mechanisms interact and work together to accelerate renal aging. This interaction is a key determinant in the onset and progression of renal diseases in the elderly population.

### 2.1 Oxidative stress

The main contributors to oxidative stress are ROS and reactive nitrogen species (RNS). Together, they are referred to as reactive oxygen and nitrogen species (RONS) (Powers et al., 2011). ROS at moderate to low concentrations are vital for a range of cellular processes, including signaling pathways, energy harvesting from organic compounds, immune responses, cell proliferation, and redox homeostasis (Bae et al., 2011). Nevertheless, when produced in excessive quantities or when interacting with other RONS, ROS cause substantial harm to cellular components (Wei et al., 2024). It is proposed that oxidative stress contributes to tissue damage and is instrumental in the emergence of aging characteristics. In fact, studies conducted on both mice and humans have demonstrated an elevation in ROS production, along with a diminished capacity for the removal of oxidants as individuals age (Vlassara et al., 2009; Miyazawa et al., 2009).

One of the contributing elements to the rise in oxidative stress is the noted decline in sirtuins, which are critical antioxidant molecules, as individuals age (Wegman et al., 2015). These deacetylases play a prominent role in mitigating kidney inflammation, fibrosis, and apoptosis, while simultaneously enhancing autophagy (Kitada et al., 2013; Yeung et al., 2004). Consequently, the diminished capacity of aged kidneys to effectively manage cellular stress may lead to observable aspects of the aging phenotype (Kume et al., 2010). Experimental mouse models exhibiting reduced expression of SIRT-1 demonstrate heightened levels of apoptosis and fibrosis following urinary tract obstruction (He et al., 2010). Additionally, the diverse functions of deacetylases encompass the deacetylation of histones and the regulation of transcription factors that are instrumental in managing cellular stress and promoting survival (Shvedunova and Akhtar, 2022).

Changes in deacetylase levels throughout aging may exacerbate the aging phenotype by impairing the kidneys' ability to counteract oxidative stress, potentially resulting in heightened oxidative DNA damage (Radak et al., 2013; Park et al., 2013). Notably, SIRT-3 has been shown to be downregulated by Angiotensin II receptor (AT2) *in vitro*, indicating that heightened levels of AT2, coupled with reduced deacetylase levels, may exert synergistic detrimental impacts on kidney function in the context of aging (Benigni et al., 2009).

### 2.2 Klotho levels decreased

Klotho, a membrane-bound protein predominantly found in the kidney, was discovered over 20 years ago for its role as an antagonist of cellular aging. In addition, it is a co-receptor of fibroblast growth factor-23 (FGF-23) (Prud’homme et al., 2022). Klotho is mainly expressed in the distal renal tubules and is crucial for maintaining the equilibrium of calcium and phosphorus balance. At the same time, it is also involved in the synthesis of calcitriol, the bioactive form of vitamin D (Fernandez-Fernandez et al., 2020). Although the exact mechanism of Klotho in aging is not yet completely elucidated. However, it is known to significantly influence a variety of aging-related pathways, including the maintenance of phosphate balance, insulin signaling, and Wnt signaling. In addition, Klotho can also affect a variety of intracellular signaling cascades, including p53/p21, cAMP, protein kinase C (PKC) and transforming growth factor-β (TGF-β) (Kuro, 2019; Quarles, 2019; Li et al., 2019; Richter and Faul, 2018).

In past studies, Klotho has been found to inhibit NFκB binding, attenuate the response of epithelial cells to oxidative stress, and improve cell survival (Sopjani et al., 2015). In animal experiments, klotho deficient mice exhibit reduced lifespan, as well as skin and muscle wasting, bone loss, and abnormal calcium deposits (John et al., 2011; Kuro-o, 2008). Conversely, mice overexpressing Klotho had an increased mean life span. In a rat model of unilateral ureteral obstruction (UUO), Klotho supplementation was found to reduce renal fibrosis (Liu Q. F. et al., 2015). The anti-fibrotic properties of Klotho are attributed to its ability to suppress FGF activity and to regulate Wnt signaling pathways (Portales-Castillo et al., 2022; Chen et al., 2022). Wnt is a signal transduction mechanism activated after injury that can promote upregulation of fibrotic genes (Tan et al., 2011). With aging, Klotho levels decrease, while Wnt signaling increases accordingly, which in turn promotes fibrosis and vascular calcification (Bian et al., 2015).

In addition, peroxisome proliferator-activated receptor-γ (PPARγ) agonists have been found to enhance Klotho expression; however, PPARγ levels decrease with aging, and therefore, Klotho expression also decreases (Wang P. et al., 2014). *In vitro* studies and aging rat models, PPARγ signaling has demonstrated a significant role in anti-oxidative stress and has some vascular enhancement effects (Zhang et al., 2008; Sung et al., 2004).

Klotho is intricately associated with oxidative stress. Some pharmacological agents with antioxidant properties have demonstrated the ability to enhance the expression of Klotho (Piao et al., 2013; Yoon and Choi, 2014; Yoon et al., 2012; Yoon et al., 2011). In contrast, research has shown that oxidative stress leads to a decrease in the levels of Klotho mRNA and protein in the inner medullary collecting duct 3 (IMCD3) cell line, which is derived from the inner medullary collecting duct of mice (Mitobe et al., 2005). Furthermore, it has been observed that increased expression of the Klotho gene correlates with a decrease in apoptotic cell count following oxidative stress-induced injury (Mitobe et al., 2005).

In a murine model of immune-mediated glomerulonephritis, increased levels of Klotho were linked to enhanced renal function, alongside a decrease in mitochondrial DNA fragmentation, superoxide anion production, lipid peroxidation, and cell death. These observations indicate that Klotho provides a protective role against oxidative stress within mitochondria (Haruna et al., 2007). The mechanism by which Klotho induces antioxidant factors and provides subsequent oxidative protection may involve a multitude of factors and pathways. Nonetheless, current research on the antioxidant properties of Klotho has primarily focused on its function in obstructing the insulin/insulin-like growth factor-1 (IGF-1) signaling pathway, as well as its role in the activation of FOXO and Nrf2 proteins. The membrane-bound form of Klotho can undergo cleavage by membrane-anchored secretases, specifically ADAM10 and ADAM17, contributing to the release of its extracellular domain into the surrounding extracellular environment.

It has been shown that the soluble form of Klotho mitigates oxidative stress by suppressing the insulin/IGF-1/PI3K/Akt/FOXO signaling cascade. This mechanism subsequently promotes the upregulation of antioxidant enzymes, such as superoxide dismutase (SOD2) and catalase (CAT). Moreover, the soluble form of Klotho significantly mitigates oxidative stress by triggering the Nrf2 cellular defense pathway. This activation stimulates the expression of genes linked to antioxidant defense mechanisms, including heme oxygenase-1 (HO-1), superoxide dismutase 2 (SOD2), catalase (CAT), and glutathione peroxidase (GPX), among others (Donate-Correa et al., 2023).

### 2.3 Cellular senescence

Repeated cellular divisions, along with the consequent attrition of telomeres, are believed to significantly contribute to the aging process. While telomere shortening is a well-studied driver of replicative senescence, other mechanisms—such as oxidative stress, DNA damage, mitochondrial dysfunction, and inflammatory signaling—can also induce senescence independently of telomere attrition (Zhang et al., 2022). As organisms age, telomeres gradually shorten, eventually reaching critically short lengths that hinder the regenerative potential of various tissues; this phenomenon is widely regarded as one of the molecular markers’ indicatives of aging.

In the human renal, telomeres exhibit a shortening rate of approximately 0.25% annually; however, there is currently insufficient data establishing a direct correlation between telomere length and any signs of renal aging in terms of histology or function (Melk et al., 2000). Short telomeres have been linked to CKD and adverse cardiovascular events, with even greater reductions observed in cases of diabetic kidney disease, where they correlate with the rate of disease progression (Raschenberger et al., 2015a; Raschenberger et al., 2015b). Furthermore, investigations involving patients undergoing dialysis have revealed an accelerated rate of telomere shortening, indicating that such changes may occur under conditions of physiological stress (Boxall et al., 2006). Nonetheless, despite these fascinating findings, the implications of telomere shortening within the framework of human aging—alongside other senescence-inducing pathways—are yet to be comprehensively understood.

As individuals age, the buildup of senescent cells becomes progressively more apparent across different organs, including the kidneys, as evidenced by the expression of markers such as p21, p16^ink4a^ or senescence-associated β-galactosidase (SA-β-Gal). While p16^ink4a^ and SA-β-Gal are widely used in aging studies, emerging evidence suggests that p21 (a cyclin-dependent kinase inhibitor) may be a more robust marker of senescence in human kidney disease, particularly in contexts of chronic injury and fibrosis (Knoppert et al., 2023; Mylonas et al., 2021). In aged animals, the regenerative response following ischemia-reperfusion injury (IRI) is significantly diminished. Renal tubular epithelial cells derived from older mice exhibit elevated levels of zinc-α2-glycoprotein (AZGP1), which has been demonstrated to suppress cell proliferation following IRI (Schmitt et al., 2008). In experiments involving AZGP1 knockout mice, a more pronounced fibrosis was observed post-IRI, while the administration of AZGP1 conferred a protective effect. This indicates that the regulation of cellular proliferation may be a critical mechanism in mitigating fibrosis during the aging process (Sörensen-Zender et al., 2015). Recent studies further highlight the role of metabolic reprogramming in tubular repair, with defective fatty acid oxidation in aged kidneys exacerbating post-IRI maladaptive repair (Mylonas et al., 2021). Although considerable research has emphasized the significance of G2/M cell cycle arrest in promoting renal fibrosis within various models of CKD, there has been a notable absence of studies specifically investigating G2/M arrest within the framework of renal aging (Yang et al., 2010). Emerging evidence now links dysregulated mTOR signaling and impaired autophagy to G2/M arrest in aged tubular cells, exacerbating senescence and fibrosis (O'Sullivan et al., 2022).

The senescence of renal tubular epithelial cells is intricately linked to the aging of the kidneys. Nevertheless, various other cell types within the renal structure may also display positive reactions to markers that signify cell cycle arrest. In a comprehensive analysis of human kidney transplant biopsies, all examined specimens demonstrated p16^ink4a^ expression that was predominantly localized within the nuclei of distal renal tubules and collecting ducts. Notably, this expression was also observed in podocytes, the epithelial cells of the glomerular wall, as well as in vascular smooth muscle cells and interstitial cells (Melk et al., 2005). Single-cell RNA sequencing of aged kidneys confirmed p16^ink4a^ enrichment in these compartments and identified a pro-senescent secretory phenotype in tubular cells (O'Sullivan et al., 2022). Nonetheless, the presence of senescent tubular epithelial cells emerged as a significant distinction between diseased and control kidneys, with such cells detected in 4/5 of diseased kidneys, in stark contrast to just 21% of normal kidneys (Sis et al., 2007).

Furthermore, findings from animal studies underscore the pivotal role of the tubular epithelium. For example, p16^ink4a^ knockout mice exhibited heightened levels of cellular senescence, accompanied by subsequent fibrosis observed in both tubular and interstitial cells, especially within the collecting ducts, after undergoing unilateral ureteral obstruction (Wolstein et al., 2010). Recent work suggests that p16^ink4a^ exacerbates mitochondrial dysfunction in aging tubules, linking senescence to metabolic dysregulation (Mylonas et al., 2021). In cases of ischemia-reperfusion injury, intranuclear p21^cip1^ was observed in both the distal and proximal nephrons, encompassing the collecting duct; however, there was no evidence of staining in the glomerulus (Megyesi et al., 2001). Furthermore, research conducted on INK-ATTAC transgenic mice has underscored the presence of senescence in proximal tubules associated with aging, particularly following the removal of senescent cells. The significance of epithelial cells is further substantiated by *in vitro* studies, which shows that various types of injuries can trigger kidney proximal tubular epithelial cells to exhibit characteristics of senescence (Jin et al., 2019).

### 2.4 Chronic inflammation

Chronic inflammation has been demonstrated to significantly contribute to renal aging. This phenomenon is marked by an increasing accumulation of macrophages and lymphocytes in the renal interstitium, which may significantly contribute to either the onset or the exacerbation of renal function decline over time. In aged kidneys, the presence of invasive macrophages and lymphocytes results in a decrease in renal mass primarily through mechanisms of tubular fibrosis and atrophy (Mei and Zheng, 2009).

The primary pathways through which chronic inflammation contributes to renal aging have been elaborated in previous studies. These pathways encompass the recruitment of inflammatory cells, which induce fibrosis *via* profibrotic factors such as interleukin-4 (IL-4), interleukin-13 (IL-13), and TGF-β. Consequently, these factors elevate the production of collagen types I and IV (Ding et al., 2001). Additionally, inflammatory cytokines like interleukin-1 (IL-1), interleukin-6 (IL-6), and tumor necrosis factor-alpha (TNF-α) promote enhanced apoptosis and the accumulation of extracellular matrix elements (Lim et al., 2012).

Chronic inflammation is associated with increased oxidative stress, which arises from the generation of ROS and advanced glycation end products (AGEs) (Chen A. et al., 2024). Moreover, it can adversely impact the proliferative capacity of stem and progenitor cells, thereby impairing the kidney’s ability to repair itself (Famulski and Halloran, 2005). Furthermore, chronic inflammation catalyzes renal senescence by precipitating cellular senescence (Ha et al., 2024). Senescent cells perpetuate damage via the senescence-associated secretory phenotype (SASP)—a pro-inflammatory secretome comprising cytokines (e.g., IL-6, TNF-α), chemokines, and matrix-remodeling factors (Basisty et al., 2020). Cells that experience senescence contribute to the exacerbation of inflammation by releasing a wide array of inflammatory factors. This process creates a harmful cycle that leads to increased fibrosis and degeneration of the renal parenchyma (Mei and Zheng, 2009). Biochemical markers that indicate cellular aging, including the cell cycle inhibitor p16, have been recognized to demonstrate an inverse relationship with renal function in individuals diagnosed with CKD as well as in those who have received kidney transplants (Melk et al., 2009; Bolignan et al., 2014).

## 3 Structural and functional changes with the aging kidney

**FIGURE 2:**
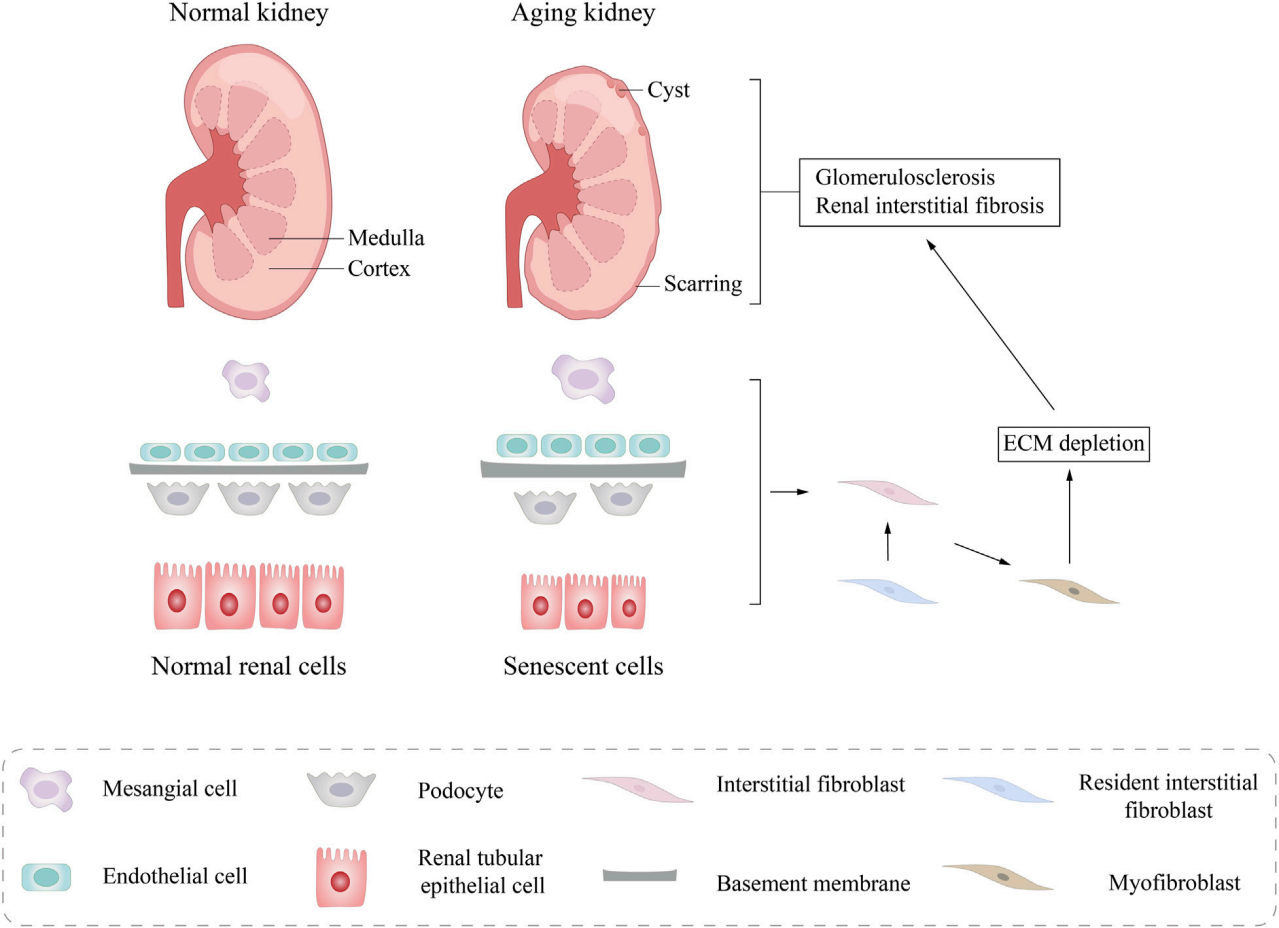
Structural changes in aging kidneys. Macroscopically, a pathological reduction in kidney size and morphological changes in kidney tissue was observed, including glomerular sclerosis, interstitial fibrosis, and tubular atrophy. Microscoisally, compensatory renal cell hypertrophy, glomerular basement membrane (GBM) thickening, podocyte loss, and tubular epithelial cell (TEC) atrophy are seen. ECM, extracellular matrix.

As individuals get older, the kidneys experience a series of anatomical transformations. Microscopically, these changes are primarily attributed to nephrosclerosis, which encompasses conditions such as arteriosclerosis, glomerulosclerosis, and glomerular atrophy accompanied by interstitial fibrosis. This pathological progression results in a diminished number of functional glomeruli, though the surviving nephrons partially compensate for this loss through hypertrophy (Figure 2).

On a macroscopic scale, there is a notable decrease in renal cortical volume as aging progresses. By the time individuals reach middle age, the renal medulla often exhibits an increased volume, while the number and size of renal cysts tend to rise significantly. As a result of these micro-and macro-anatomical alterations, people may experience functional degenerative changes ranging from mild to severe. Normal aging leads to a progressive reduction of the glomerular filtration rate (GFR), but such changes do not occur in all individuals. Although declining, older adults often maintain clinically meaningful GFR levels in the absence of CKD (Denic et al., 2016a).

### 3.1 Structural changes of the aging kidney

Based on various research methodologies, the changes in structure observed in aging kidneys can be classified into two primary types: microanatomical changes derived from renal biopsy findings, and macroanatomical changes determined through imaging examinations, including CT scans.

#### 3.1.1 Micro-anatomical changes

Renal biopsy primarily identifies nephrosclerosis and nephron hypertrophy. The key characteristics of nephrosclerosis encompass focal and global glomerulosclerosis, tubular atrophy, interstitial fibrosis, and arteriosclerosis. Arteriosclerosis—characterized by fibrous thickening and/or intimal hyalinization of renal vasculature—is believed to induce nephron ischemia, which subsequently causes global glomerulosclerosis, accompanied by tubular atrophy and fibrosis (Hommos et al., 2017). Glomerular ischemia results in the fibrosis of the capsule, which in turn causes the capillary clusters to wrinkle and the basement membrane to thicken progressively. When the balance between the production and breakdown of the glomerular extracellular matrix is disrupted, the space of Bowman gradually accumulates a matrix-like, translucent material. Ultimately, this process culminates in the collapse of the glomerular plexus, giving rise to the formation of globally sclerotic glomeruli (GSG) (Martin and Sheaff, 2007).

Numerous studies have demonstrated that conditions such as obesity, diabetes, and certain surgical procedures can result in nephron hypertrophy, primarily characterized by the enlargement of both glomeruli and tubules. As various comorbidities tend to become more pronounced with advancing age, research often reports larger glomeruli in deceased patients. (Tsuboi et al., 2013a; Tsuboi et al., 2013b; Tsuboi et al., 2012). Nevertheless, research focusing specifically on healthy living kidney donors has not shown a significant rise in glomerular volume related to the aging process (Denic et al., 2017; Denic et al., 2016b; Tan et al., 2010). While nephron hypertrophy, which refers to the enlargement of non-sclerosed glomeruli (NSG) volume and tubular area alongside a reduction in glomerular density, shows a relatively weak correlation with age, it exhibits a stronger association with specific coexisting conditions that are prevalent among older populations, such as obesity, diabetes, and hyperuricemia (Elsherbiny et al., 2014).

In addition to that, the quantity of nephrons declines as individuals age (Shankland et al., 2023). However, the reduction in nephron count cannot be solely ascribed to age-related glomerulosclerosis; in fact, less than one-third of this decline can be linked to GSG (Hommos et al., 2017).

#### 3.1.2 Macro-anatomical changes

CT scans indicate that age-related changes in the kidneys are predominantly marked by a decrease in renal cortical volume, accompanied by the formation of benign cysts and tumors (Muzaale et al., 2024). Furthermore, it has been observed that the average number of nephrons decreases by 48% from the ages of 18–29 to 70–75, while cortical volume diminishes by only 16% (Denic et al., 2017). This discrepancy can be attributed to a compensatory increase in medullary volume, which persists until around the age of 50 (Wang et al., 2014b). The observed loss of GSG volume, and the corresponding tubular atrophy, may explain the reduction in renal cortical volume associated with aging.

In addition, GSG may promote the formation of renal cysts. With aging, there are more and more tubular diverticula, which may be a prodromal lesion of renal cysts caused by aging of the kidneys (Larsen et al., 2018). Parenchymal cysts in the kidney cortex and medulla are similar, with higher frequency and larger volume in the elderly. In addition, the incidence of parapelvic cysts and angiomyolipoma also increased with age (Rule et al., 2012).

### 3.2 Functional changes of the aging kidney

According to previous studies, renal aging is related to changes in renal function, mainly including changes in GFR, renal tubular reabsorption and secretion capacity, and renal endocrine function (Zhou et al., 2008a). Specifically, the glomerular filtration capacity, and thus the ability to clearly metabolize waste and excess fluid from the body, decreases (Waas et al., 2021). However, it is not completely clear whether the decreased glomerular function is due to natural aging or concomitant complications (Zhou et al., 2008b). Due to the aging of renal tubules, the elderly are more prone to hypovolemia and drug-induced hyperkalemia (Kielstein et al., 2003; Sands, 2012). In addition, with aging, the production and release of renin is reduced, so the total aldosterone level is also reduced, and the renin-angiotensin system (RAS) within the kidney and throughout the body is suppressed (Karam and Tuazon, 2013). Together, these alterations in kidney function due to aging may raise the likelihood of developing kidney disease.

#### 3.2.1 Glomerular function

At birth, the GFR is relatively low, gradually rising to reach adult levels by the end of the second year of life. This rate typically stabilizes at around 140 mL/min/1.73 m^2^ until approximately 30 years of age. After this point, the GFR begins to decline almost linearly, decreasing by roughly 8 mL/min/1.73 m^2^ for each subsequent decade (Waas et al., 2021). However, the question remains contentious as to whether the observed decline in GFR is attributable to the natural aging process or to concurrent comorbidities (Zhou et al., 2008b). In a systematic review of 129359 patients from 1958 to 2021, the annual rate of decline in GFR ranged from −0.37 to −1.07 mL/min/1.73 m2/year in healthy adults without hypertension (Guppy et al., 2024).

Under normal circumstances, renal vasodilation induces a noteworthy elevation in renal blood flow and the GFR, indicating the renal hemodynamic and functional reserve’s capacity. Nonetheless, in healthy elderly individuals, the simultaneous infusion of amino acids alongside dopamine has been observed to cause an improtant decrease in both the maximum renal plasma flow (RPF) and the GFR (Fuiano et al., 2001; Esposito et al., 2007). This decrease in kidney hemodynamics and functional reserve may compromise the kidneys' adaptive responses to acute ischemia, consequently heightening the risk of AKI among the elderly population.

#### 3.2.2 Tubular function

Aging is linked to notable alterations in renal tubular function (Li et al., 2024). Older adults exhibit a heightened sensitivity to hypovolemia compared to their younger counterparts. In instances of dietary sodium chloride deficiency, the reduction in urinary sodium excretion occurs at a significantly slower rate in the elderly (Yeung et al., 2021). Nonetheless, under stable conditions, both older and younger individuals can maintain sodium balance effectively. Research has demonstrated that the proximal fractional sodium reabsorption is markedly greater in the elderly; however, this is counterbalanced by a reduction in distal fractional sodium reabsorption (Fliser et al., 1997).

Additionally, older adults are particularly vulnerable to drug-induced hyperkalemia, attributed to the active transport of urinary potassium within the distal renal tubules and collecting ducts, a process that is closely linked to sodium reabsorption mediated by the aldosterone-regulated Na-K ATPase transporter. Moreover, with advancing age, the kidneys' capacity to concentrate and dilute urine decreases, leading to a higher frequency of nocturia, an increased risk of dehydration and hypernatremia, and a heightened vulnerability to hyponatremia when there is an excessive intake of fluid (Sands, 2003; Faull et al., 1993).

Older adults exhibit a heightened vulnerability to drug toxicity, primarily attributable to alterations in pharmacokinetics resulting from diminished renal function and the decline of virtually all other organ systems (Christopher et al., 2022). Additionally, the aging process induces noteworthy alterations in body composition, marked by a reduction in total body water and a simultaneous rise in body fat (Yu et al., 2024). Furthermore, aging adversely influences the pharmacodynamics of numerous medications; for instance, the reduced responsiveness of the cardiovascular system to stimulation by β-epinephrine in older individuals makes them more susceptible to orthostatic hypotension when undergoing treatment with antihypertensive agents. Consequently, it is imperative to initiate pharmacological treatment in elderly patients with the lowest effective dose, with the possibility of gradually titrating the dosage upwards as deemed necessary (Zhou et al., 2008b).

#### 3.2.3 Endocrine function

With advancing age, there is a notable decline in kidney function, which results in a reduced synthesis of erythropoietin (EPO) by the kidneys. This physiological change could be a contributing factor to a heightened incidence of anemia among the elderly (Ble et al., 2005; Eisenstaedt et al., 2006). Even though serum EPO levels tend to increase as age advances in healthy individuals, an intriguing observation is that anemic older adults exhibit lower EPO levels compared to their younger counterparts facing anemia. This disparity suggests a diminished physiological response to declining hemoglobin levels in older adults (Ferrucci et al., 2007).

Moreover, the kidneys serve as the primary site for the clearance of insulin from systemic circulation, a process that occurs through glomerular filtration as well as proximal tubular uptake and degradation (Duckworth et al., 1998). Consequently, diminished kidney function in older adults is linked to impaired insulin clearance. In addition to this, older adults commonly experience heightened peripheral insulin resistance (Basu et al., 2003). Insulin secretion is also compromised within the aging population, characterized by a diminished secretory reserve of β cells. Therefore, while older adults experience a decrease in systemic insulin clearance compared to their younger counterparts, it is paradoxical that they are at a heightened risk of developing insulin resistance.

Furthermore, the kidneys are integral to the regulation of sympathetic nervous activity. An increase in sympathetic nerve tone, which may result from decreased GFR and other physiological factors, is probable to lead to the promotion of atherosclerosis among the elderly demographic (Zhou et al., 2008b).

## 4 Aging in kidney disease

**FIGURE 3:**
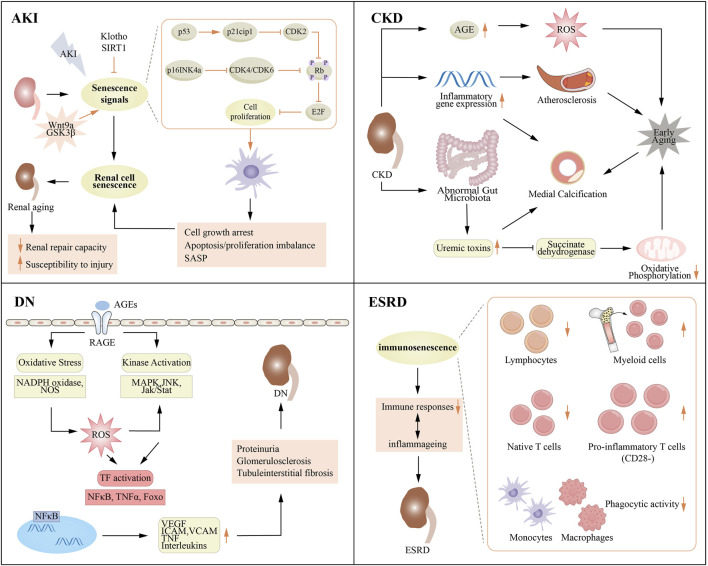
Association between aging and various renal diseases. AGE, advanced glycation end product; AKI, acute kidney injury; CKD, chronic kidney disease; DN, diabetic nephropathy; ESRD, end stage renal disease; ICAM, intercellular cell adhesion molecule; JNK, c-JunN-terminal kinase; MAPK, mitogen-activated protein kinase; NADPH, nicotinamide adenine dinucleotide phosphate; TNF, tumor necrosis factor; VCAM, vascular cell adhesion molecule; VEGF, vascular cell adhesion molecule.

The link between aging and kidney disease is receiving increasing attention because it not only affects the quality of life of individuals but may also affect disease progression and treatment response (Figure 3). AKI is defined by an abrupt decline in renal function, which can arise from various factors, including severe infections, surgical interventions, and exposure to drugs or toxins. The risk of developing AKI tends to increase with age, as the renal reserve capacity diminishes in older adults, who are often burdened by chronic diseases (Chang-Panesso, 2021). CKD encompasses a range of chronic and progressive disorders that ultimately lead to irrevocable damage to kidney structure and function. Aging contributes greatly to in the progression of CKD; the kidneys' ability to filter and repair diminishes over time, rendering the elderly more vulnerable to the onset of CKD (Zhang et al., 2024). DN, a common complication associated with diabetes mellitus, results in renal impairment. While DN can develop at any age, older individuals may exhibit heightened susceptibility to this condition due to a history of inadequate glycemic control and the presence of multiple comorbidities (Narasimhan et al., 2021). ESRD represents the final stage of CKD, marked by a near-total loss of renal function. Aging serves as distinct risk factor for the progression to ESRD, as older adults frequently experience a more rapid decline in kidney function and exhibit a less favorable response to therapeutic interventions. However, some studies have shown that the rate of decline in renal function slows down with age, suggesting that other factors are at play, particularly the increased risk of death and the differences in underlying causes of chronic kidney disease among elderly patients (Conway et al., 2009).

### 4.1 Acute kidney injury

The incidence of chronic diseases such as hypertension, diabetes, atherosclerosis, and heart failure tend to rise with advancing age, which subsequently leads to the phenomenon of polypharmacy among the elderly population. This situation elevates the risk of adverse events. Furthermore, it is estimated that about 1/5 of AKI cases are linked to the use of nephrotoxic medications, with the prevalence soaring to as high as 66% in the elderly (Wang et al., 2014c). In this demographic, cardiovascular medications and non-steroidal anti-inflammatory drugs (NSAIDs) are frequently employed to manage related conditions, yet they can potentially compromise renal function through mechanisms such as fluid depletion or drug interactions (Ribeiro et al., 2022).

AKI following coronary angiography remains a clinically significant concern, particularly in elderly patients. While historically termed “contrast-induced nephropathy” (CIN), recent evidence suggests that the direct causative role of contrast media may have been overestimated, with AKI often attributable to concurrent risk factors such as hemodynamic instability, heart failure, or preexisting renal dysfunction. Notably, AKI in 20% of older angiography patients, with heart failure—not contrast exposure—emerging as the strongest predictor of renal injury. This distinction underscores the concept of contrast-associated AKI (CA-AKI), where contrast administration coincides with but does not necessarily induce renal injury. In an emergency setting, concerns about renal function should not be viewed as a barrier, and risk reduction, such as rehydration, and minimizing the volume of contrast material, should be emphasized (Proctor et al., 2025).

An interesting observation from Hollenberg’s research revealed that older patients demonstrated a diminished vasodilatory response to acetylcholine, whereas their vasoconstrictive response to angiotensin remained unchanged. This indicates that older adults might be more susceptible to AKI during periods of hypoperfusion, as they display a weakened vasodilatory capacity combined with an intact, or potentially augmented, vasoconstrictive response (Hollenberg et al., 1974). In the study by Kang et al., decreased vascular endothelial growth factor (VEGF) expression and increased thrombospondin-1 (TSP-1) levels were found in the kidneys of aged rats, which may lead to decreased repair capacity and thus impaired angiogenic response. This could explain the decreased glomerular and peritubular capillaries and the marked decrease in endothelial cell proliferation in aged rats (Kang et al., 2001).

Furthermore, a study conducted by the TRIBE-AKI Consortium involving 1444 patients undergoing cardiac surgery showed that postoperative VEGF and placental growth factor (PGF) levels were inversely linked to AKI risk and mortality, whereas VEGR levels were positively associated with AKI and mortality (Mansour et al., 2019). The results indicate that the formation of new blood vessels on the surface plays a crucial part in the kidney’s healing process. Furthermore, the decrease in VEGF levels associated with aging kidneys might be a significant contributor to the heightened incidence and death rates of AKI observed in older individuals.

More and more studies have indicated that cell senescence plays a pivotal role in both the onset and progression of AKI, and inhibiting cell senescence can facilitate the recovery of kidney function. For example, in a model of sepsis-induced AKI, lipotoxin A4 was used to inhibit the senescence of renal tubular cells, and renal function was recovered (Chen et al., 2021). Similarly, in the context of contrast-induced AKI, pretreatment with paclitaxel effectively diminished cellular senescence and mitigated tissue damage (Bae et al., 2020). Notably, individuals aged over 70 are at a markedly elevated risk of developing AKI, a condition that has been linked to immunosenescence and a reduction in the levels of α-Klotho protein expression (Li and Lerman, 2020). Additionally, aging appears to compromise the regenerative potential of renal cells, thus prolonging recovery from AKI stemming from ischemia-reperfusion injury.

AKI is typically associated with the activation of the DNA damage response (DDR), which involves the activation of ataxia telangiectasia mutation (ATM) and ataxia telangiectasia and activation of Rad3-related (ATR) proteins. This response contributes to the production of cell cycle inhibitors, specifically p21^Waf1/Cip1^, resulting in the arrest of the cell cycle within renal tubular epithelial cells (Kellum and Chawla, 2016). The activation of ATR serves a protective role for renal tubules by alleviating inappropriate repair mechanisms and decreasing the level of fibrosis (Kishi et al., 2019). Furthermore, the production of ROS is linked to impaired mitochondrial function, which subsequently triggers the p53/p21^Cip1^ signaling pathway (Bhargava and Schnellmann, 2017). This cascade contributes to DNA damage and further cell cycle arrest (Takemura et al., 2020). The Klotho protein is crucial in the functionality of renal tubular epithelial cells, and mice deficient in Klotho exhibit traits associated with aging (Andrade et al., 2018). In the context of AKI, the activation of the p16^Ink4a^ pathway can exacerbate cellular senescence, leading to G2/M phase cell cycle arrest. This mechanism forms a “vicious cycle” that may provoke pathological alterations in adjacent cells through the secretion of the SASP (Jin et al., 2020; Lin et al., 2022).

Research has demonstrated that the elimination of senescent cells can be beneficial for maintaining kidney function. For example, Mylonas et al. reported significant reductions in cystatin C levels—a sensitive marker of renal function—following senescent cell clearance in murine fibrosis models, indicating meaningful functional preservation alongside attenuated fibrosis (Mylonas et al., 2021). However, cellular senescence may also exhibit a protective effect in the context of AKI, as evidenced by certain small-molecule inhibitors which can induce proximal tubule cell-cycle arrest that have been found to improve kidney injury outcomes in murine models (DiRocco et al., 2014).

### 4.2 Chronic kidney disease

According to the latest KDIGO (Kidney Disease: Improving Global Outcomes) 2024 guidelines, CKD is defined as abnormal kidney structure or function, lasting at least 3 months, which has an impact on health. The specific manifestations were any of the following abnormalities lasting for more than 3 months: glomerular filtration rate (GFR) < 60 mL/min/1.73 m^2^; Renal injury markers included albuminuria (UACR≥30 mg/g), abnormal urine sediment, renal tubule-related lesions, histological abnormalities, structural abnormalities found on imaging, history of renal transplantation, and persistent hematuria. The guidelines classify CKD stages based on GFR and albuminuria levels as follows: GFR levels are divided into six stages: G1 (GFR≥90 mL/min/1.73 m^2^), G2 (GFR 60–89 mL/min/1.73 m^2^), G3a (GFR 45–59 mL/min/1.73 m^2^), G3b (GFR 30–44 mL/min/1.73 m^2^), G4 (GFR) 15–29 mL/min/1.73 m^2^) and G5 (GFR<15 mL/min/1.73 m^2^ or dialysis required). The level of albuminuria was divided into three grades: A1 (UACR<30 mg/g), A2 (UACR 30–299 mg/g), and A3 (UACR≥300 mg/g) (KDIGO, 2024, 2024).

CKD is acknowledged as similar to diseases associated with aging, with the age-related SASP playing a significant role in its progression (Schroth et al., 2020). In the context of CKD, immunosenescence is marked by a higher ratio of terminally differentiated T cells, telomere shortening in monocytes, reduced thymic output, and a diminished capacity to eliminate senescent kidney cells. Additionally, CKD is linked to hyperphosphatemia, which triggers senescence in myoblasts, endothelial cells, and vascular smooth muscle cells, consequently fostering sarcopenia and vascular calcification. Together, these elements are crucial contributors to the advancement of CKD (Crépin et al., 2020).

Senescent cells play an important role in the occurrence and development of CKD. Acute injury (such as oxidative stress, ischemia, *etc.*) can induce early cell senescence by activating p16^ink4a^ and p21^Cip1^ pathways, which is initially manifested as temporary cell cycle arrest to promote DNA repair. However, sustained injury leads to irreversible cell cycle arrest, ultimately promoting the transition from AKI to CKD. Cellular senescence is a common mechanism for AKI-CKD transition and progression of various glomerular diseases, and targeting senescent cells or related pathways may be a potential therapeutic strategy (Zhang et al., 2024). The mechanisms underlying cellular senescence associated with CKD involve factors such as hyperphosphatemia and the buildup of uremic toxins. These factors lead to the senescence of various cell types, including myoblasts, endothelial cells, and smooth muscle cells, thereby exacerbating conditions such as sarcopenia and vascular calcification (Sosa et al., 2018; Olmos et al., 2017; Troyano et al., 2015). In the initial stages of CKD, a decrease in renal reabsorption of inorganic phosphorus (Pi) results in an elevated secretion of fibroblast growth factor 23 (FGF-23), which facilitates the renal secretion of Pi. As the disease progresses to stage 3, systemic hyperphosphatemia typically arises, further exacerbating systemic inflammation and vascular calcification, and consequently accelerating the process of early vascular aging (Carracedo et al., 2019). Elevated levels of phosphate adversely affect endothelial function and activate the pro-inflammatory nuclear factor kappa B (NFκB) pathway (Abbasian et al., 2015; Voelkl et al., 2018).

Furthermore, compromised renal function results in the buildup of azotemic substances, which in turn promotes chronic inflammation. This inflammatory state hinders immune activation and serves as a pivotal element in the initiation of immunosuppression (Glorieux et al., 2020). In CKD, a variety of circulating pro-inflammatory markers, including IL-6, fetuin A, and tumor necrosis factor (TNF), exhibit progressive alterations as kidney function deteriorates. These changes are intricately linked to both renal aging and the disease process itself (Thomas et al., 2022). The stimulation of inflammasome signaling pathways, stimulated by cytokines, ROS, and damage-associated molecular patterns (DAMPs), can lead to increased levels of IL-1 and interleukin-18 (IL-18). Such elevations disrupt the activity of epigenetic regulators, including microRNAs (miRNAs), which are crucial for maintaining cellular homeostasis during aging. Additionally, the heightened production of pro-inflammatory cytokines and chemokines from senescent cells exacerbates the inflammatory load and interferes with the aging process in CKD. Moreover, oxidative stress has been demonstrated to inhibit Klotho expression by activating miR-200c. In parallel, the dysregulation of TGF signaling ultimately contributes to Klotho deficiency and renal fibrosis by inducing the expression of various miRNAs (Morii et al., 2019). This disruption leads to a dysregulation between pro-inflammatory and anti-inflammatory macrophages, coupled with mitochondrial damage, which ultimately results in chronic systemic uremic inflammation that is a hallmark of CKD (Ebert et al., 2020).

Two protein-bound uremic toxins, indoxyl sulfate (IS) and p-cresyl sulfate (pCS), are resistant to removal via conventional dialysis. These uremic toxins can significantly induce inflammatory responses, oxidative stress, and damage to vascular endothelial cells, all of which lead to the decline in kidney function and the exacerbation of related comorbidities (Ramezani and Raj, 2014; Sirich et al., 2014; Muteliefu et al., 2012). In cases of CKD, AGEs accumulate and function as uremic toxins. This accumulation results in mitochondrial dysfunction, an elevation in ROS, and structural changes due to macromolecular cross-linking. As a result, the AGE axis enhances oxidative stress and inflammation in the renal cytoplasmic environment, contributing to cellular and tissue damage. This damage accelerates the early aging of proximal tubular epithelial cells and mesangial cells (Chaudhuri et al., 2018; Stinghen et al., 2016; Liu W. J. et al., 2015). Uremic toxins exert unique and detrimental effects on various biological processes, including the balance of pro-oxidative and anti-oxidative factors, mitochondrial function, inflammation, and ultimately lead to cellular senescence. These processes are recognized as key characteristics of the aging process (López-Otín et al., 2013).

The role of autophagy in CKD is complex, with bidirectional regulatory effects that may either play a protective role or promote disease progression, depending on the disease stage and cell type. In proximal renal tubular epithelial cells (TECs), autophagy inhibition, such as chloroquine or ATG7 depletion, reduces fibrosis, suggesting that autophagy may promote injury (Nam et al., 2019). In distal TECs, activation of autophagy, such as upregulation of LC3-II/ATG7 and inhibition of AKT-mTOR, had anti-fibrotic effects, suggesting a protective role (Nam et al., 2019). Autophagy is activated after acute injury but decreases in the chronic course and may be associated with disease progression. Inhibition of autophagy (as in the aging kidney) may accelerate age-related nephropathy. Excessive activation of autophagy may lead to renal tubular atrophy and fibrosis in some cases, but it can also exert anti-fibrotic effects by degrading excess collagen (Zhang et al., 2024).

Although there is still controversy over the use of uniform criteria to define diseases in the elderly, studies have clearly shown that in the elderly population, the risk of adverse outcomes increases with the increase in the stage of CKD (Delanaye et al., 2019; Grams et al., 2023). Current guidelines use a sustained GFR below 60 mL/ minute/ 1.73 m^2^ for more than 3 months (corresponding to GFR classes G3a-G5) as the threshold for the diagnosis of CKD, which is indeed significantly lower than the average for healthy young men and women. However, since a marked decline in the GFR in young adults often accompanies other markers of kidney injury, such cases can still be diagnosed promptly (KDIGO, 2024, 2024). Although both cross-sectional and longitudinal studies confirm that mean GFR declines with age, there is significant individual variation - not everyone has a precipitous decline in kidney function with age (Grams et al., 2023).

### 4.3 Diabetic nephropathy

Around 1/3 of people diagnosed with type 1 diabetes and nearly 1/2 those with type 2 diabetes will develop diabetic nephropathy (DN) at some stage in their lives. DN is characterized by the thickening of both tubular and glomerular basement membranes, the merging and loss of podocyte foot processes, as well as the proliferation of the mesangial matrix (Thomas et al., 2015; Alicic et al., 2017).

Prior research has suggested that the pathogenesis of DN involves the interplay of various metabolic and hemodynamic factors, including hyperglycemia, AGEs, and the renin-angiotensin system (RAS) (Cao et al., 2025; Tang et al., 2022; Psyllaki and Tziomalos, 2024). These elements are intricately associated with the activation of ROS generation, which is mediated by protein kinase C (PKC) and subsequently results in the activation of the downstream transcription factor, NF-κB (Ming et al., 2022).

Both renal aging and DN display multiple common pathophysiological mechanisms, particularly the buildup of AGEs. This accumulation instigates oxidative stress, inflammatory responses, and promotes a pro-aging phenotype (Zgutka et al., 2023). Numerous studies have documented elevated markers of cellular aging in biopsy and nephrectomy samples, which coincide with manifestations of DN. Within the renal tubules, markers such as senescence-associated β-galactosidase (SA-β-GAL) and p16 are found to have a direct correlation with blood glucose levels. Meanwhile, the expression of p16 in the glomeruli is linked to the occurrence of proteinuria associated with DN.

Research focusing on individuals with type 2 diabetes has indicated that the progression of DN correlates with heightened levels of DNA damage. Senescent kidney tubular cells are implicated in the advancement of CKD in diabetic animal models treated with streptozotocin (S et al., 2010). Strategies aimed at reducing high glucose levels by employing sodium-glucose cotransporter-2 inhibitors (SGLT2i) have been shown to significantly lessen the onset of cellular senescence, potentially attributed to the synthesis of β-hydroxybutyrate (Fang et al., 2021; Kulkarni et al., 2022). Furthermore, the complement system is actively involved in DN pathogenesis by modifying DNA methylation patterns in renal tubular cells. Histone deacetylase (HDAC) inhibitors, such as valproic acid, have demonstrated efficacy in countering the upregulation of complement C5a receptors instigated by diabetes, thereby preventing cellular senescence and diminishing functional impairment associated with DN (Coughlan et al., 2022).

Telomere shortening is a widely recognized occurrence in both type 1 and type 2 diabetes, and this process may be hastened by factors such as inflammation, hyperglycemia, glycation end products, and persistent oxidative stress (Sutanto et al., 2019; O'Donovan et al., 2011; Carrero et al., 2008; Houben et al., 2008; Kurz et al., 2004). The loss of telomeres may instigate stress-induced premature aging and is linked to renal-cell senescence, proteinuria, and the advancement of DN (Fyhrquist et al., 2010; Sampson and Hughes, 2006). Studies indicate that high blood sugar levels are a primary contributor to the accelerated aging of renal tubular cells, a process that is closely linked to telomere shortening. Furthermore, the telomeres of white blood cells in individuals suffering from diabetic nephropathy also exhibit significant shortening. This telomere shortening holds potential as a diagnostic indicator for diabetic nephropathy. Moreover, extensive population studies have shown a significant correlation between the reduction in telomere length and the advancement of nephropathy (Mazidi et al., 2017).

An increasing body of literature indicates that epigenetic modifications contribute substantially to the progression of DN, primarily by activating mechanisms related to oxidative stress. For instance, research utilizing a rat model of diabetes has demonstrated that histone H3K27 methylation acts to suppress the expression of the antioxidant repressor TXNIP. Notably, inhibiting the enzyme EZH2 exacerbates symptoms such as proteinuria, podocyte dysfunction, and renal oxidative stress (Siddiqi et al., 2016). Furthermore, hyperglycemia has been shown to enhance TXNIP expression by modulating histone acetylation and methylation processes (De Marinis et al., 2016). In podocytes, elevated glucose levels can also provoke promoter hypomethylation and hyperacetylation of histone H3, leading to increased expression of the oxidative stress regulator p66Shc, while simultaneously recruiting specific methyltransferases to diminish the levels of superoxide dismutase SOD2 (Paneni et al., 2012; Bock et al., 2013; Zhong and Kowluru, 2013).

Moreover, epigenetic changes are pivotal in sustaining chronic inflammation and impairing autophagy, both of which are fundamental mechanisms associated with cellular aging (Sánch et al., 2023; Shu et al., 2023). In vascular endothelial cells and inflammatory cells, hyperglycemia induces modifications of H3K4me1 mediated by the SET7 histidine methyltransferase. This process subsequently amplifies the expression of the pro-inflammatory factor NF-κB (Mohammed et al., 2025). Current research highlights the significance of non-coding RNAs in the pathogenesis of DN. In studies involving db/db mice, it has been found that miRNA-125b functions to inhibit H3K9me3 by downregulating the Suv39h1 histone methyltransferase. This inhibition subsequently results in the upregulation of chemokines associated with monocytes and lymphocytes, including MCP-1 and IL-6 (Chen Z. et al., 2024). Another non-coding RNA, miRNA-146a, is particularly elevated early in the disease process, serving to protect the kidney from inflammation and fibrosis by suppressing the activation of M1 macrophages (Bhatt et al., 2016).

Furthermore, hyperglycemia induces the activation of histone deacetyltransferase 4 (HDAC4) within podocytes. This activation contributes to the deacetylation of STAT1, which consequently results in the inhibition of autophagy (Wang et al., 2014d). Finally, the application of the histone deacetylase inhibitor troamine-A has been shown to mitigate kidney injury by preserving the levels of the anti-aging protein Klotho (Lin et al., 2017). Collectively, these studies underscore the essential significance of epigenetic modifications in relation to cellular aging and DN.

Mitophagy is crucial for sustaining healthy mitochondria and serves a protective function by eliminating damaged mitochondria and protein aggregates (Liang and Kobayashi, 2016; Aggarwal et al., 2016; Diot et al., 2016). In diabetic conditions, more than 50% of renal tubular cells display mitochondrial fragmentation, which is accompanied by a marked increase in mitochondrial reactive oxygen species (mtROS) within the renal cortex (Zhan et al., 2015; Guo et al., 2015). This observation suggests a significant impairment of mitophagy in DN. The process of mitophagy relies on kinase signaling pathways, with PTEN-induced putative kinase 1 (PINK1) being the most critical. After cellular injury occurs, PINK1 transmits signals to the cytosolic E3 ubiquitin ligase referred to as Parkin. In turn, Parkin enhances the mitophagy signal by promoting the PINK1-mediated recruitment of optineurin (OPTN) and NDP52 (Wauer et al., 2015; Lazarou et al., 2015). OPTN possesses a ubiquitin-binding domain that enables it to recognize and bind to polyubiquitinated cargoes, thus aiding their transportation for the formation of autophagosomes (Wong and Holzbaur, 2015). Notably, a loss of Parkin expression is correlated with a shortened lifespan, while Parkin overexpression is associated with an extension of lifespan (Leduc-Gaudet et al., 2022). Similarly, the reduction of PINK1 leads to a shortened lifespan and speeds up the aging process (Scialò et al., 2016).

Recent findings intriguingly suggest that OPTN plays a role in the senescence of renal tubular epithelial cells when exposed to elevated glucose levels (Chen et al., 2018). A multitude of researches have illustrated that mitophagy is compromised in the kidneys of diabetic individuals, which is linked to mitochondrial dysfunction, an overproduction of mitochondrial ROS, and a diminished expression of PINK1 and Parkin (Xiao et al., 2017). Podocytes demonstrate a significantly elevated baseline rate of autophagy as they age. Nevertheless, this increased autophagic activity becomes compromised in the presence of diabetes, both in living organisms and under elevated glucose conditions in laboratory settings (Fang et al., 2013). This dysfunction contributes to cellular damage, glomerular injury, and the advancement of kidney disease. Therefore, it can be inferred that deficiencies in mitophagy may precipitate early senescence across various renal cell types, thereby further exacerbating the advancement of kidney disease in diabetic environments.

Numerous reports indicate that uremic waste can contriute to mitochondrial dysfunction and hypermethylation of the Klotho gene, both of which are pivotal in the senescence of DN cells (Sun et al., 2017; Young and Wu, 2012). AGEs, recognized as uremic toxins in DN, are implicated in various pathways contributing to cellular senescence. Specifically, AGEs have been shown to exert deleterious effects that promote aging phenotypes across multiple organ systems within the human body, including the kidneys (Semba et al., 2010). Research has demonstrated that AGEs can trigger cellular senescence via several mechanisms, including the initiation of p21 in an oxidative stress-dependent manner, upregulation of p16 expression, inhibition of autophagy by diminishing PINK1/Parkin activity, and the promotion of inflammation in renal cells (Liu et al., 2014; Liu J. et al., 2015; Yamagishi et al., 2015; Zha et al., 2017; Shi et al., 2019; Chen et al., 2016).

### 4.4 End-stage renal disease

End-stage renal disease (ESRD) is characterized by a markedly higher likelihood of mortality resulting from cardiovascular events and infections. Additionally, it leads to significant structural and functional impairments in multiple organ systems, with a pronounced impact on the cardiovascular, immune, and musculoskeletal systems (Wouk, 2021). This condition predominantly afflicts the elderly, with the typical age for starting renal replacement therapy being 62.3 years for males and 63.4 years for females. Notably, the number of ESRD patients receiving treatment has reached over 15,000 annually within the age group of 70–79 years. The predominant pathology seen in ESRD is glomerulosclerosis, which is intricately linked to factors such as aging, diabetes, hypertension, and other glomerular conditions. Indeed, glomerulosclerosis is prevalent in more than 70% of individuals aged 40 years and older, and its incidence tends to escalate with advancing age (Wiggins, 2012). Moreover, numerous phenotypic similarities exist between the manifestations of ESRD and the aging process itself (Kooman et al., 2017).

In non-uremic mice, the loss of the NF-κB subunit 1 initiates a chain reaction of chronic inflammation, which in turn accelerates telomere shortening and results in a progressively aging phenotype (Jurk et al., 2014). Among dialysis patients, a pronounced trend of “telomere reduction” associated with various inflammatory markers has been observed when compared to control groups of the same age (Carrero et al., 2008; Costello-White et al., 2015; Kooman et al., 2014). Oxidative stress, a key contributor to the process of biological senescence, is notably elevated in individuals with ESRD and is closely linked to the presence of uremic inflammation (Steyers and Miller, 2014; Zewinger et al., 2016). Additionally, uremic inflammation disrupts “nutrient perception,” a significant indicator of aging (López-Otín et al., 2013). Cytokines such as TNF and IL-6 promote catabolic pathways by activating the ubiquitin-proteasome system while concurrently inhibiting anabolic processes through IGF resistance and irregular mTOR regulation (Kooman et al., 2014; Stenvinkel et al., 2005). Moreover, systemic inflammation correlates with a decrease in the population of endothelial progenitor cells in uremic patients, which may adversely impact vascular repair mechanisms. Kramann et al. have indicated that Gli1+ cells, which are essential adventitial precursor cells, could function as a hopeful target for lessening arterial calcification (Kramann et al., 2016). The process of cellular aging may further render cells increasingly susceptible to damage induced by urotoxins and oxidative stress (Carracedo et al., 2013).

Furthermore, the slow deterioration in renal function is linked to a range of alterations within the adaptive immune system, collectively referred to as premature immunosenescence. This occurrence significantly affects the death rate and illness frequency in ESRD patients (Betjes, 2020). The memory T cell pool in patients with ESRD experiences varying degrees of alteration, characterized primarily by a general reduction in the absolute counts of CD4 T cells, which can largely be credited to a decline in naive and central memory T cell populations (Gruver and Sempowski, 2008). Both CD4 and CD8 T cell subsets demonstrate a linear decrease in telomere length with advancing age, with ESRD patients exhibiting significantly shorter telomeres compared to healthy individuals. Research indicates that the T cell receptor (TCR) repertoire in ESRD patients is skewed due to the selective growth of certain T cell subsets, and T cells in these patients show reduced proliferation in uremic serum (Huang et al., 2017; Huang et al., 2015). Moreover, there is a considerable decrease in the population of naive B cells within the circulating B cell pool of patients with ESRD, and these cells exhibit heightened vulnerability to apoptosis. Nevertheless, the processes that lead to the maturation of B cells into plasma cells capable of secreting immunoglobulins remain to be comprehensively explored (Pahl et al., 2010).

ESRD also impacts the overall quantity and functionality of dendritic cells (DCs), contributing to decreased DC density in the skin, thereby impairing their capacity to present antigens to T cells within lymph nodes (Verkade et al., 2004). The ongoing deterioration of renal function negatively impacts the genesis and preservation of naive T cells, as well as the differentiation pathways essential for memory T cell development. Consequently, this results in a decrease in the number of naive T cells and an expansion of a highly differentiated, pro-inflammatory memory T cell subset with a restricted diversity of T cell receptors. Moreover, naive T cells exhibit a heightened tendency toward apoptosis and display age-associated impairments in critical intracellular phosphorylation pathways. The observed alterations are consistent with the concept of accelerated aging in the T cell compartment, indicating that the immune characteristics of patients with ESRD might show signs of aging that are approximately 20 years ahead compared to those with healthy kidney function (Betjes et al., 2011).

Uremia is linked to increased oxidative stress and inflammation, which exacerbate one another. Conditions such as intestinal perforations and low-grade infections-exemplified by periodontitis-further aggravate this pathological process. The thymus gland exhibits heightened vulnerability to inflammatory responses and oxidative stress, which exacerbates the typical atrophy associated with aging (Venet and Monneret, 2018; Majumdar et al., 2019). Additionally, the bone marrow’s production of lymphoid precursors is impaired, leading to fewer new T cells and a swifter reduction in circulating naive T cells. This reduction could limit the diversity of the T-cell receptor library and promote the development of memory T cells into a highly differentiated, pro-inflammatory state. Prior cytomegalovirus infections may have caused a significant rise in CD28-negative T cells, which are further increased in ESRD patients. The growth of these memory T cells can occupy key immune spaces in the bone marrow, impeding the maturation of other T cells and thus weakening the adaptive immune system. Meanwhile, this condition promotes systemic inflammation, which increases the likelihood of infections, cancer, and cardiovascular incidents (Betjes, 2020).

## 5 Aging and kidney transplantation

Human studies have revealed a notable association between the abundance of senescent cells in the kidney before transplantation and the later emergence of interstitial fibrosis, tubular atrophy, and chronic allograft nephropathy (CAN) following transplantation (Parimon et al., 2023; Fu et al., 2019; Mai et al., 2022). Both experimental animal and human studies indicate that elevated mRNA expression levels of p21^cip1^ and p16^ink4a^ in pre-transplant biopsy samples are linked to suboptimal transplant outcomes (Melk et al., 2009; McGlynn et al., 2009). Within the domain of kidney transplantation (KT), the application of immunosuppression may contribute to diminished immune activation and an increase in senescent cell accumulation, potentially due to impaired immune clearance mechanisms (Hoffmann et al., 2015). Moreover, the interplay between senescent cells and the immune environment following transplantation is particularly complex. Notably, allograft rejection often occurs alongside an increase in cellular senescence. Additionally, elevated expression levels of glomerular, tubular, and interstitial cells originating from rejected allografts are associated with differing degrees of chronic allograft nephropathy (Vavrinec et al., 2024).

Senescent cells are likely pivotal in elucidating the influence of age on graft outcomes. In human instances, kidneys sourced from older donors exhibit a higher count of senescent cells, which may impede the transplanted kidney’s capacity to recover from injury (Kishi et al., 2024). Consequently, these grafts are more vulnerable to ischemic damage and demonstrate a greater propensity for delayed graft function post-transplantation (Oberhuber et al., 2012; Lim et al., 2013). The impact of aging on transplant outcomes was further confirmed in kidney transplants from p16^ink4a^ knockout (KO) mice. In these studies, recipient mice exhibited reduced fibrosis, significantly improved graft survival rates, diminished tubulointerstitial injury, and elevated scores for tubular epithelial proliferation. These results underscore the notion that the buildup of senescent cells could significantly contribute to the pathological alterations seen after kidney transplants. Accordingly, therapeutic approaches aimed at targeting senescent cells may potentially enhancing long-term graft survival and function (Braun et al., 2012).

## 6 Targeting aging for the treatment of kidney diseases

Managing AKI in older adults necessitates a thorough evaluation of their general health and any additional health conditions. Treatment usually involves identifying and correcting the cause of AKI, such as medications, infection, cardiac insufficiency, or dehydration. Supportive treatment measures are key, including the maintenance of appropriate blood pressure, glycemic control, adequate hydration, and nutritional support. Avoiding nephrotoxic drugs, such as nsaids and certain antibiotics, is also important. If necessary, the dose of the drug being used may need to be adjusted to reduce the burden on the kidneys.

Regarding the treatment of CKD and ESRD, the uremic environment is associated with premature aging, and dialysis and KT may theoretically improve the premature aging phenotype. However, multiple factors during dialysis, such as poor biocompatibility, contaminated dialysis water, and immunosuppressive therapy, lead to proinflammatory and prooxidative effects that may adversely affect the aging process (Kooman et al., 2014). Following KT, patients face an increased vulnerability to various complications, notably ischemia-reperfusion injury and rejection, which accelerate aging and are reflected in measures such as telomere shortening (Sturmlechner et al., 2017). In addition, immunosuppressive state may lead to residual senescent cells in the transplanted kidney, transmitting pro-aging signals. Thus, dialysis and KT do not prevent the aging process and may even accelerate the onset of the aging phenotype (Childs et al., 2015). Consequently, the management of CKD and ESRD in elderly patients should involve a thorough evaluation of the individual’s overall health conditions, life expectancy, quality of life, and personal preferences. Current research indicates that sodium-glucose cotransporter 2 (SGLT-2) inhibitors and glucagon-like peptide-1 (GLP-1) receptor agonists show good efficacy and high safety in elderly patients with CKD (Hu et al., 2024). For patients with ESRD, the selection of renal replacement therapy should consider multiple factors rather than solely relying on age or cognitive status; in addition to traditional hemodialysis and kidney transplantation, conservative renal management based on individualized needs should also be included in the treatment decision-making (Bhandari et al., 2022). It is worth noting that with the development of digital medical technology, innovative means such as wearable monitoring devices and virtual reality technology are expected to provide new support for the disease management of elderly CKD patients (Canaud et al., 2023).

Managing DN in older adults involves a holistic approach to decelerate kidney disease progression and lower the likelihood of cardiovascular issues. Treatment typically involves tight glycemic and blood pressure control, as well as angiotensin-converting enzyme (ACE) inhibitors or angiotensin II receptor blockers (ARBs) to mitigate the advancement of nephropathy. However, for those with decreased renal function, it is crucial to control the dose of drugs to avoid harm caused by drug accumulation. Multiple drug classes show nephroprotective potential in CKD, with benefits observed in DN. These therapeutic options encompass RAS blockade, SGLT2 inhibition, GLP-1 receptor activation, endothelin-1 receptor blockade (atrasentan), vasopressin V2 receptor antagonism (tolvaptan), and selective mineralocorticoid receptor antagonism (finerenone), all demonstrating differential efficacy in retarding renal function decline (Huang et al., 2023). Senolytics can selectively eliminate senescent cells and have emerged as a promising therapeutic strategy. The combination treatment of dasatinib (D) and Quercetin (Q) for diabetic nephropathy is currently under investigation, but the long-term safety and dosage regimens still need to be verified (Hickson et al., 2019). A recent study has described the pathological mechanism by which histone crotonylation modification regulates renal fibrosis, and proposed a potential therapeutic strategy: by inhibiting the activity of ACSS2 and reducing histone crotonylation, thereby affecting the IL-1β-mediated activation of macrophages and the aging process of renal tubular cells, in order to achieve the goal of alleviating renal fibrosis (Li et al., 2024).

Regular surveillance of kidney function and proteinuria levels is essential to evaluate the effectiveness of treatment and to adjust treatment. For elderly patients with kidney disease, in terms of daily life, healthy diet, limiting sodium and protein intake, appropriate daily water intake, weight control, regular exercise, smoking and drinking cessation are also very beneficial for maintaining kidney health and alleviating disease progression.

## 7 Conclusion

This manuscript addresses the mechanisms of renal aging including oxidative stress, reduced Klotho levels, cellular senescence, as well as chronic inflammation. These mechanisms are intricately linked and together lead to changes in kidney structure and function. Structurally, the main manifestations are nephrosclerosis and hypertrophy of the nephron. Functionally, it is mainly manifested as the reduction of GFR, renal tubular reabsorption and secretion capacity, and the change of renal endocrine function. As a result, the risk of developing various kidney diseases—such as AKI, CKD, DN, and ESRD—escalates significantly with advancing age.

In summary, acquiring a thorough knowledge of the mechanisms and determinants linked to renal aging is crucial for the prevention and management of age-related renal diseases. Continued research is imperative to delve deeper into the molecular pathways underlying renal aging and to devise innovative therapeutic approaches aimed at enhancing renal health and enhancing the overall wellbeing of the elderly.
